# Molecular Survey of Hemopathogens in Dogs, Including Blood Donors, from Central-Western Brazil

**DOI:** 10.3390/pathogens14111180

**Published:** 2025-11-18

**Authors:** João Vitor dos Santos Alves da Silva, Lorena Freitas das Neves, Maria Eduarda Bolzan, Liliane Maria do Rosario Batista, Francisco Anilton Alves Araujo, Rosangela Zacarias Machado, Marcos Rogério André

**Affiliations:** 1 Vector-Borne Bioagents Laboratory (VBBL), Department of Pathology, Reproduction and One Health, School of Agrarian and Veterinary Sciences (FCAV), São Paulo State University (UNESP), Jaboticabal Campus, Jaboticabal 14884-900, SP, Brazil; joao-vitor.silva@unesp.br (J.V.d.S.A.d.S.);; 2 OHV One Health Veterinary—Centro de Hemoterapia Pet, Brasília 71736-501, DF, Brazil; lilianebatistabmv@gmail.com (L.M.d.R.B.); centrodehemoterapiapet@gmail.com (F.A.A.A.)

**Keywords:** *Babesia vogeli*, *Bartonella* spp., ‘*Candidatus* Mycoplasma haematoparvum’, canine blood donation, *Ehrlichia canis*, *Mycoplasma haemocanis*

## Abstract

Blood transfusions are indispensable in Veterinary Medicine, providing therapeutic support in cases of hematological disorders. Several pathogens can cause disease and/or exacerbate the condition of immunocompromised dogs or those requiring a transfusion. This study aimed to investigate the molecular occurrence of hemopathogens (*Bartonella* spp., *Ehrlichia* spp., *Anaplasma* spp., piroplasmids, and hemoplasmas) in blood donor and patient dogs using samples from a clinical veterinary laboratory in Brazil. One hundred blood samples were collected from each group. All dogs tested negative for *Bartonella* spp. in all performed assays. Among the 100 dogs from the clinical veterinary laboratory, 15% (95% CI: 9.3–23.3) tested positive for *Ehrlichia* spp., 6% (95% CI: 2.8–12.5) for *Anaplasma* spp., 3% (95% CI: 1.0–8.5) for *Babesia* spp., and 2% (95% CI: 0.6–7.0) for hemoplasmas. Blood donor dogs tested positive for hemoplasmas (5%) (95% CI: 2.2–11.2). Additional conventional and real-time PCR assays followed by sequencing confirmed the presence of *Ehrlichia canis*, *Anaplasma platys*, *Babesia vogeli*, ‘*Candidatus* Mycoplasma haematoparvum’, and *Mycoplasma haemocanis*. The molecular detection of *E. canis*, *A. platys*, ‘*Ca*. M. haematoparvum’, and *M. haemocanis* in dogs from midwestern Brazil reinforces the relevance of molecular tools in diagnosing hemopathogens. This is the first molecular detection of hemoplasmas in canine blood donors from Brazil. This finding indicates their silent circulation and highlights the importance of molecular screening to prevent the worsening of clinical conditions and the risk of turning recipients into new sources of infection.

## 1. Introduction

The understanding of transfusion medicine and the use of blood components has been progressively increasing, leading to a broader application in veterinary medicine [1]. Anemic conditions are the main indications for blood transfusion in veterinary practice and may result from blood loss due to hemorrhage, red blood cell destruction, or insufficient erythrocyte production [2]. Although blood transfusion is a highly important tool to promote clinical improvement in anemic patients during the pursuit of a definitive diagnosis [3], it often lacks complete safety in its application. The high demand for blood transfusions, combined with the impossibility of some dogs to donate blood components, frequently contributes to blood shortages in veterinary medicine [4,5]. Due to emergency situations and this shortage, clinical and surgical routines often require blood transfusions without proper screening for hemopathogens. It is essential to conduct appropriate screening panels to detect microorganisms and reduce the risk of pathogen transmission [6].

Transfusion reactions can occur after a blood transfusion between dogs. The transmission of infectious disease-causing agents represents one of the causes of non-immunological transfusion reactions [7]. The adoption of screening tests for infectious agents is extremely important in veterinary medicine to minimize the risk of disease transmission and progression. In this context, vector-borne agents, such as *Anaplasma* spp., *Babesia* spp., *Bartonella* spp., *Ehrlichia* spp., *Hepatozoon* spp., *Leishmania* spp., hemotropic *Mycoplasma* spp., *Neorickettsia* spp., *Rickettsia* spp., and *Trypanosoma* spp., must be included in screening protocols. Minimum and ideal standards for the quality of blood to be transfused should be based on geographic location, donor origin, test availability, cost, and emergencies, always aiming to identify and pre-screen canine blood donors to maintain safe blood banks [8]. In Brazil, there are currently no specific or mandatory regulations for canine blood donation, which further reinforces the need for research aimed at establishing appropriate screening protocols to ensure more safety in transfusion practices.

To the best of our knowledge, no study has reported the detection of *Bartonella* spp. or hemoplasmas in canine blood donors in Brazil, although *Babesia vogeli*, *Anaplasma platys*, and *Ehrlichia canis* have been previously reported in donor candidates [9]. Few studies have investigated the occurrence of hemopathogens in canine blood donors. In the United States, Stegeman et al. (2003) [10] detected *Babesia gibsoni* (Piroplasmorida: Babesiidae) DNA in a female dog that developed mild fever, splenomegaly, and thrombocytopenia after receiving a blood transfusion from a donor dog infected with this hemoparasite. In South Korea, *Mycoplasma haemocanis* DNA was detected in an immunosuppressed dog that had received a blood transfusion from a donor infected with the agent [11]. Antibodies *anti*-*Anaplasma phagocytophilum* and *Ehrlichia canis* (Rickettsiales: Anaplasmataceae) were detected in 4.7% and 1.3%, respectively, of blood donor candidate dogs (*n* = 150) in Italy [12]. Among 6140 canine blood bags, *A. phagocytophilum* (0.065%), *Mycoplasma haemocanis* (0.77%), ‘*Candidatus* Mycoplasma haematoparvum’ (Mycoplasmatales: Mycoplasmataceae) (0.29%), and *Bartonella* spp. (Hyphomicrobiales: Bartonellaceae) (0.081%) were detected in Canada [13]. It has been shown that *M. haemocanis* DNA remains in primary canine erythrocytes for at least 29 days during storage, with a higher DNA copy number/µL observed on day 29 compared to day 1 in blood bags stored at 4–8 °C, emphasizing the potential risk of transmission of this pathogen to dogs following transfusion [14].

The investigation of pathogens capable of causing disease in animals is of great importance, as they can have a significant impact on both animal and human health, thereby encompassing the broader One Health concept, since approximately 61% of human infectious diseases are of zoonotic origin [15,16].

Vector-borne diseases represent major public health challenges, given their significant increase in recent decades, mainly due to socioeconomic, environmental, and climatic changes. Accordingly, to achieve better long-term solutions, it is extremely important to conduct research within the One Health framework, involving professionals such as veterinarians, entomologists, and parasitologists [17].

We hypothesized that dog blood donors might be more likely to harbor *Bartonella* spp. and hemoplasmas, as these agents are not routinely evaluated in donor screening tests. Conversely, *Babesia* spp. and *Ehrlichia* spp. would be expected less frequently, since dogs with anemia and trombocytopenia (the primary hematological manifestations of infection) would likely have been excluded from donation. Clinically evaluated dogs without prior screening could present a broader variety of hemopathogens, reflecting a higher risk of infection.

This study aimed to investigate the presence of hemopathogens (such as *Bartonella* spp., *Ehrlichia* spp., *Anaplasma* spp., *Babesia* spp., and hemoplasmas) in apparently healthy canine blood donors compared to those submitted for routine clinical pathology examinations, either for preventive evaluation or investigation of possible clinical alterations and regardless of clinical suspicion, in order to better understand the risk of transmission of blood-borne agents through blood transfusion in dogs in the Brazilian Central-West region.

## 2. Materials and Methods

### 2.1. Ethics Committee

The procedures adopted for this study were approved by the Ethics Committee on the Use of Animals of the School of Agricultural and Veterinary Sciences (FCAV/UNESP), under protocol number 3970/22.

### 2.2. Study Site

The blood samples used in this study were obtained from a commercial clinical pathology laboratory and blood bank located in Brasilia, Federal District (Central-Western Brazil). A total of 200 blood samples were collected: 100 samples (55 males and 45 females) from dogs registered as blood donors, and the remaining 100 samples (59 males and 41 females) from animals submitted for routine clinical examinations, either for preventive evaluation or investigation of possible clinical alterations, regardless of clinical suspicion.

Dogs registered as blood donors by the blood bank were subjected to screening protocols at each collection, including clinical evaluation, complete blood count, serum biochemistry (urea, creatinine, and total protein), and serological testing (SNAP^®^ 4Dx^®^ Plus Test—IDEXX Laboratories, Westbrook, ME, USA) (detection of antibodies to *Anaplasma phagocytophilum*, *Anaplasma platys*, *Ehrlichia canis*, *Ehrlichia ewingii*, *Borrelia burgdorferi*, and *Dirofilaria immitis* antigens). Inclusion criteria for this group included clinically healthy presentation, hematologic and biochemical values within reference ranges, and seronegativity for the abovementioned vector-borne pathogens. Hematological parameters of the blood donor dogs were within the reference ranges described by Feldman et al. (2000) [18].

Between January and March 2023, approximately two milliliters of blood from dogs were provided by convenience by a commercial clinical pathology laboratory and a veterinary blood bank, both located in Brasília: the researchers had no direct contact with the sampled animals. The samples were then stored in RNase- and DNase-free cryotubes and transported in a liquid nitrogen container to the Vector-Borne Bioagents Laboratory (VBBL, UNESP/FCAV—Jaboticabal Campus, SP), where they were kept at –80 °C until processing.

The sample size of 100 dogs per group was determined by the number of animals available during the sampling period and to maintain uniformity between groups, as including a larger number of donors was not feasible at the collection site during this timeframe. Although no formal sample size calculation was performed, this number reflects the routine availability of canine blood donors and clinical patients within the study period.

### 2.3. DNA Extraction from Canine Blood Samples

DNA extraction from the blood samples was performed using the BIOPUR Mini Spin Plus Extraction Kit (BP101-250, Mobius Life Science, Pinhais, PR, Brazil), following the manufacturer’s instructions. DNA samples were evaluated for purity (260/280 and 260/230 ratios) and quantity (concentration) using a spectrophotometer (NanoDrop 2000/2000c, Thermo Scientific, Waltham, MA, USA). DNA samples from dogs available in our laboratory were used as positive controls. Microtubes containing ultrapure sterile water were used as negative controls during the DNA extraction procedures.

### 2.4. Conventional PCR (cPCR) for the Endogenous Mammalian Gene Glyceraldehyde-3-Phosphate Dehydrogenase (gapdh)

To verify the absence of PCR inhibitors in the DNA samples extracted from canine blood aliquots, a conventional PCR (cPCR) targeting the endogenous mammalian glyceraldehyde-3-phosphate dehydrogenase (*gapdh*) gene was performed [19] (Supplementary Material S1). Ultrapure sterile water was used as a negative control.

### 2.5. Detection of Bartonella *spp.*

#### 2.5.1. *Bartonella* spp. Culture

The pre-enrichment liquid medium used was based on the previously described *Bartonella*-Alpha-proteobacteria Growth Medium (BAPGM). For this, 500 mL of IPL-41 Insect Medium) was supplemented with 0.055 mg of NAD, 0.69 mg of NADP, 1.1 mg of ATP, 1.1 mg of sodium pyruvate, and 1.1 g of yeast extract (Sigma-Aldrich, St. Louis, MO, USA). Amino acid supplementation was performed by adding 35.1 mg of L-arginine HCl, 8.66 mg of L-cystine HCl, 11.64 mg of L-histidine, 14.6 mg each of L-isoleucine and L-leucine, 20.14 mg of L-lysine, 4.16 mg of L-methionine, 9.02 mg of L-phenylalanine, 13.22 mg of L-threonine, 2.7 mg of L-tryptophan, 12 mg of L-tyrosine 2Na 2H_2_O, and 13 mg of L-valine (Dinâmica, Indaiatuba, São Paulo, Brazil). The pH of the medium was then adjusted to 6.2 and subsequently sterilized through a 0.2 µM filter (Corning, Corning, NY, USA). After filtration, the medium was supplemented with 55 mL of defibrinated ovine blood (previously tested negative for *Bartonella* spp. by qPCR) and adjusted to a final concentration of 11% *v*/*v*. Aliquots of 200 µL of canine blood were added to 2 mL of medium containing defibrinated ovine blood, placed in 20 cm^2^ culture flasks (model 430639, Corning, Corning, NY, USA), and incubated for 7 days at 35 °C with 5% CO_2_ under constant agitation in a CO_2_/O_2_ Water Jacketed incubator (NuAire, Plymouth, MN, USA) [20,21].

After seven days, 200 µL of the liquid culture were subjected to DNA extraction using the BIOPUR Mini Spin Plus Extraction Kit (BP101-250), following the manufacturer’s recommendations. Additionally, 200 µL of the content from each culture flask were plated on chocolate agar enriched with VX supplement (containing NAD, hemin, and growth factors) (Laborclin, Pinhais, Paraná, Brazil) and incubated at 37 °C with 5% CO_2_ for up to 60 days in a CO_2_/O_2_ Water Jacketed incubator (NuAire, Plymouth, MN, USA), with daily monitoring. Colonies suggestive of bacterial growth were harvested and subjected to DNA extraction using the boiling method [22].

Both the liquid culture aliquots (BAPGM) and suggestive *Bartonella* colonies (gray-whitish, slightly translucent, smooth, and circular) were subjected to qPCR assays for *Bartonella* spp., targeting the 16–23S rRNA intergenic region (ITS) [23,24]. No solid cultures on chocolate agar were performed directly from the blood samples; instead, the samples were maintained in BAPGM for 7 days prior to plating on chocolate agar.

#### 2.5.2. Quantitative Real-Time PCR (qPCR) for *Bartonella* spp.

DNA samples extracted directly from aliquots of canine blood, from liquid cultures after seven days of incubation, and from suspect colonies on chocolate agar were subjected to a generic qPCR assay for *Bartonella* spp., targeting the 16–23S rRNA intergenic spacer (ITS) region [23,24] (Supplementary Material S1). DNA samples were analyzed in duplicate, and when the difference in quantification cycle (Cq) between replicates exceeded 0.5 then the samples were reanalyzed in triplicate. Ultrapure sterile water was used as a negative control. Serial dilutions (from 2.0 × 10^7^ to 2.0 × 10^0^ copies) of a gBlock containing a 226 bp fragment of the ITS region of *Bartonella henselae* (Integrated DNA Technologies, Coralville, IA, USA) were used as positive controls, as well as for standard curve construction. The copy number was calculated using the following formula:

(Xg/μL DNA/[plasmid length in bp × 660]) × 6.22 × 10^23^ = plasmid copies/μL.

All analyses were performed according to the MIQE guidelines (“Minimum Information for Publication of Quantitative Real-Time PCR Experiments”). Amplification efficiency (E) was calculated based on the slope of the standard curve using the formula: E = 10^(−1/slope) [25]. qPCR assays were performed using the C1000-CFX96 thermal cycler (Bio-Rad^®^, Hercules, CA, USA), and the data were visualized and stored using CFX Manager software, version 2.1. (Bio-Rad^®^, Hercules, CA, USA).

### 2.6. Molecular Detection and Characterization of Piroplasmids

DNA samples extracted from dog blood were subjected to a nested PCR (nPCR) targeting an ~800 bp fragment of the 18S rRNA gene for piroplasmids [26]. Positive samples were further analyzed by PCR targeting six molecular markers: 18S rRNA (~1500 bp) (Greay et al., 2018; Kawabuchi et al., 2005) [27,28], *hsp70* (~740 bp) (Soares et al., 2011) [29], *cox*-1 (924 bp) (Corduneanu et al., 2017) [30], *cox*-3 (~600 bp) (Barbosa et al., 2019; Schreeg et al., 2016) [31,32], *cytB* (~1000 bp) (Barbosa et al., 2019; Schreeg et al., 2016) [31,32], and ITS2 (~590 bp) (Duarte et al., 2008) [33] (Supplementary Material S1). DNA samples of *Theileria equi* [34] and *Babesia bigemina* [35] were used as positive controls for the reactions. Ultrapure sterile water was used as a negative control for the reactions.

### 2.7. Molecular Detection and Characterization of Ehrlichia *spp.* and Anaplasma *spp.*

DNA from canine blood samples was analyzed via multiplex real-time PCR (qPCR) targeting the *groEL* gene (83 bp) for *Ehrlichia* spp. and *Anaplasma* spp. [36]. Samples positive for *Ehrlichia* spp. were further tested using qPCR targeting the *dsb* gene (378 bp) of *E. canis*, followed by a conventional PCR (409 bp) [37]. Samples positive for *Anaplasma* spp. were subjected to a nested PCR targeting the 16S rRNA gene (546 bp) [38] (Supplementary Material S1). The samples subjected to the qPCR assay were tested in duplicates. DNA from *Anaplasma platys* [39] and the Jaboticabal strains of *E. canis* and *Anaplasma marginale* were used as positive controls. Ultrapure sterile water was used as a negative control.

### 2.8. Molecular Detection and Characterization of Hemoplasmas

DNA extracted from dog blood samples was subjected to a qPCR targeting the 16S rRNA gene of hemoplasmas [40] (Supplementary Material S1). Reactions were run in duplicates with triplicate confirmation when Cq differences exceeded 0.5. Amplification efficiency was calculated using the following formula: E = 10^(−1/slope) [25]. Serial dilutions (1.0 × 10^7^ to 1.0 × 10^0^ copies) of a gBlock containing a 259 bp fragment of the *Mycoplasma haemofelis* 16S rRNA gene (IDT, Coralville, IA, USA) were used for curve construction and as positive controls. Nuclease-free sterile water (Promega, Madison, WI, USA) was used as a negative control. A melting curve (Tm) analysis was performed [40] to differentiate hemoplasma species. qPCR-positive samples were then additionally subjected to: (i) semi-nested PCR targeting the 16S rRNA gene (~1107 bp) (Di Cataldo et al., 2020) [41]; (ii) PCR assay targeting the 16S rRNA gene (~1380 bp) (Maggi et al., 2013) [42]; (iii) PCR targeting the 23S rRNA gene (~800 bp) (Mongruel et al., 2020) [43] (Supplementary Material S1). DNA from *Mycoplasma ovis* (Mongruel et al., 2020) [43] was used as a positive control; ultrapure sterile water was used as a negative control.

### 2.9. Agarose Gel Electrophoresis

PCR products were subjected to 1.0% horizontal agarose gel electrophoresis stained with ethidium bromide (0.5 μL/mL) in TBE buffer pH 8.0 (44.58 mM Tris-base, 0.44 mM boric acid, 12.9 mM EDTA), run at 90 V/150 mA for 60 min. A 100 bp DNA ladder (Life Technologies^®^, Carlsbad, CA, USA) was used, and results were visualized using a UV transilluminator (ChemiDoc MP Imaging System, Bio-Rad^®^, Hercules, CA, USA). Data were stored using Image Lab software, version 5.2.1 (Bio-Rad^®^, Hercules, CA, USA).

### 2.10. Sequencing and Phylogenetic Analyses

PCR products were purified using ExoSAP-IT™ PCR Product Cleanup Reagent (Thermo Scientific, San Jose, CA, USA) according to manufacturer instructions. DNA quantification was performed using a NanoDrop spectrophotometer.

Sequencing was conducted using the Sanger chain termination method (Sanger et al., 1977) [44] on an ABI PRISM 3700 DNA Analyzer (Applied Biosystems, Foster City, CA, USA) at the Human Genome and Stem Cell Research Center (Institute of Biosciences—USP, São Paulo, Brazil).

Chromatogram analysis was performed using BioEdit v.7.0.5.3 (Hall, 1999) [45], evaluating the quality of peaks corresponding to each base. Consensus sequences were assembled using Phred-Phrap version 23 (Ewing & Green, 1998 [46]; Ewing et al., 1998) [47], using both forward and reverse reads with a minimum base quality score of 20, and analyzed using BLAST (Altschul et al., 1990) [48], via the NCBI web server to compare with sequences previously deposited in GenBank (http://www.ncbi.nlm.nih.gov/genbank, accessed on 4 May 2025).

Sequences were saved in FASTA format and aligned with homologous sequences using Clustal/W (Thompson et al., 1994) [49], via BioEdit. Alignments in FASTA format were converted to Nexus format using the Alignment Transformation Environment (Glez-Peña et al., 2010) [50],. Phylogenetic inference was conducted using the Maximum Likelihood method with W-IQ-Tree software (http://iqtree.cibiv.univie.ac.at/, accessed on 20 August 2025, web server) (Nguyen et al., 2015 [51]; Trifinopoulos et al., 2016 [52]). Clade support was assessed using bootstrap analysis with 1000 replicates (Felsenstein, 1985) [53]. Phylogenetic trees were edited and rooted (via outgroup) using TreeGraph 2.13.0 beta (Stöver & Müller, 2010) [54].

### 2.11. Comparison of Molecular Occurrence of Hemopathogens Among Dog Groups and Sex

The prevalence of each hemopathogen (*Anaplasma* spp., *Babesia* spp., *Bartonella* spp., *Ehrlichia* spp., and *Mycoplasma* spp.) was estimated, and corresponding 95% confidence intervals were calculated using the Wilson method. Differences in pathogen prevalence between dog groups (dog blood donors and dogs undergoing routine clinical examinations) and between sexes (males vs. females) were assessed using Fisher’s exact test due to the binary nature of the outcome and the presence of zero counts in some contingency tables. Statistical significance was defined as *p* ≤ 0.05.

The analyzes were performed using the software R Project version 4.5.2 (https://cran.r-project.org/bin/windows/base/, accessed on 3 November 2025).

## 3. Results

### 3.1. Microbiological and Molecular Investigation of Bartonella *spp.*

All DNA samples obtained from liquid cultures (*n* = 200) and from the dogs’ blood (*n* = 200) tested negative in the quantitative PCR (qPCR) for *Bartonella* spp. No colonies suggestive of *Bartonella* spp. were isolated after plating aliquots of the liquid cultures onto chocolate agar.

### 3.2. Molecular Detection of Piroplasmids

Among the 100 samples from dogs that underwent routine clinical pathology testing, 3 (3/100—3%) (95% CI: 1.0–8.5) tested positive in the nested PCR (nPCR) for piroplasmids based on the 18S rRNA gene. After additional PCR assays for molecular characterization and sequencing, two sequences were obtained for the 18S rRNA gene, three for the *cox-1* gene, and two for the *hsp70* gene.

BLASTn analysis revealed that the obtained sequences shared identities ranging from 98.69% to 100% with *Babesia vogeli* sequences detected in dogs from China, Brazil, the USA, and Japan (Table 1).

All blood samples from dogs registered as blood donors tested negative in the screening nested PCR for piroplasmids.

### 3.3. Molecular Detection of Ehrlichia *spp.* and Anaplasma *spp.*

Of the 100 samples from the group of dogs that underwent routine clinical pathology testing, 15 (15%) (95% CI: 9.3–23.3) were positive for *Ehrlichia* spp. and 6 (6%) (95% CI: 2.8–12.5) for *Anaplasma* spp. in a multiplex real-time PCR (qPCR) assay targeting the *groEL* gene. The Cq values obtained in the qPCR reactions ranged from 25.25 to 38.82 for *Ehrlichia* spp. and from 34.29 to 38.74 for *Anaplasma* spp.

All 15 samples positive for *Ehrlichia* spp. in the multiplex qPCR (*groEL*) assay were also positive in a qPCR for *E. canis* based on the *dsb* gene. One sequence was obtained from the PCR targeting the *dsb* gene of *Ehrlichia* spp., which after BLASTn analysis showed 100% identity with an *E. canis* sequence obtained from *Rhipicephalus linnaei* from Australia (MT005829) (Table 1). Two out of six positive samples for *Anaplasma* spp. in the multiplex qPCR were also positive in the nested PCR (nPCR) assay targeting the 16S rRNA gene. BLASTn analysis of these sequences showed 100% identity with an *Anaplasma platys* sequence identified in a dog from South Africa (MK814414) (Table 1).

All samples from the blood donor group were negative in the multiplex qPCR assay targeting *Ehrlichia* spp. and *Anaplasma* spp. based on the *groEL* gene.

**Figure 1:**
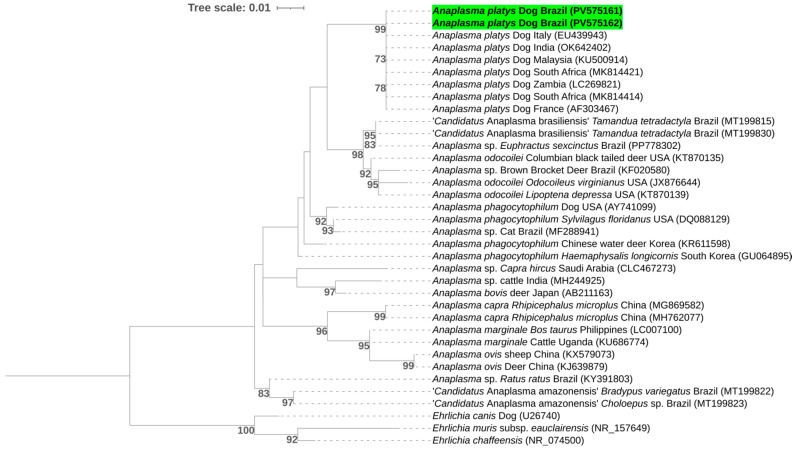
Phylogenetic tree inferred by the Maximum Likelihood method and the GTR + G evolutionary model based on an alignment of 332 pb bp of the 16S rRNA gene. Sequences obtained in this study are highlighted in green. Bootstrap values below 70% were omitted. Sequences of *Ehrlichia muris* subsp. *eauclairensis*, *Ehrlichia chaffeensis*, and *Ehrlichia canis* were used as outgroups.

Phylogenetic analysis, inferred by the Maximum Likelihood method using the GTR + G evolutionary model and based on alignment of a 332 pb bp fragment of the 16S rRNA gene, positioned the obtained sequences within the same subclades as previously detected *A. platys* sequences from South Africa (MK814414 and MK814421), Zambia (LC269821), France (AF303467), Italy (EU439943), Malaysia (KU500914), and India (OK642402) (Figure 1).

Coinfection with *Anaplasma* sp. and *Babesia* sp. was detected in one dog that underwent routine clinical pathology testing.

### 3.4. Hemoplasmas

Of the 100 samples from the group of dogs submitted for clinical pathology examinations, 2 (2%) (95% CI: 0.6–7.0) tested positive in the qPCR for hemoplasmas based on the 16S rRNA gene. Among the 100 samples from the group of dog blood donors, 5 (5%) (95% CI: 2.2–11.2) tested positive in the qPCR for hemoplasmas.

All samples that showed to be positive in the qPCR protocol also tested positive in the semi-nested PCR, the conventional PCR targeting the 16S rRNA gene, and the conventional PCR targeting the 23S rRNA gene. In the group of dogs that underwent clinical pathology testing, two high-quality sequences were obtained for the 16S rRNA gene and one for the 23S rRNA gene of hemoplasmas. In the blood donor group, four readable 16S rRNA gene sequences and one 23S rRNA gene sequence were obtained.

Following BLASTn analysis, the obtained sequences showed identity values ranging from 99.52% to 100% with ‘*Candidatus* Mycoplasma haematoparvum’ and from 99.77% to 100% with *Mycoplasma haemocanis* (Table 1). The mean Cq values obtained in the qPCR assays ranged from 24.57 to 27.07. The positive samples had average quantifications ranging from 236.1 copies/µL to 1909 copies/µL, with standard deviation values ranging from 7.77 to 152.9. The melting temperature (Tm) values of the samples were 77 °C for three samples (corresponding to ‘*Candidatus* Mycoplasma haematoparvum’), 77.5 °C (corresponding to ‘*Candidatus* M. haematoparvum’) for one sample, 78 °C (corresponding to *M. haemocanis*) for two samples, and 78.5 °C (corresponding to *M. haemocanis*) for one sample.

**Figure 2:**
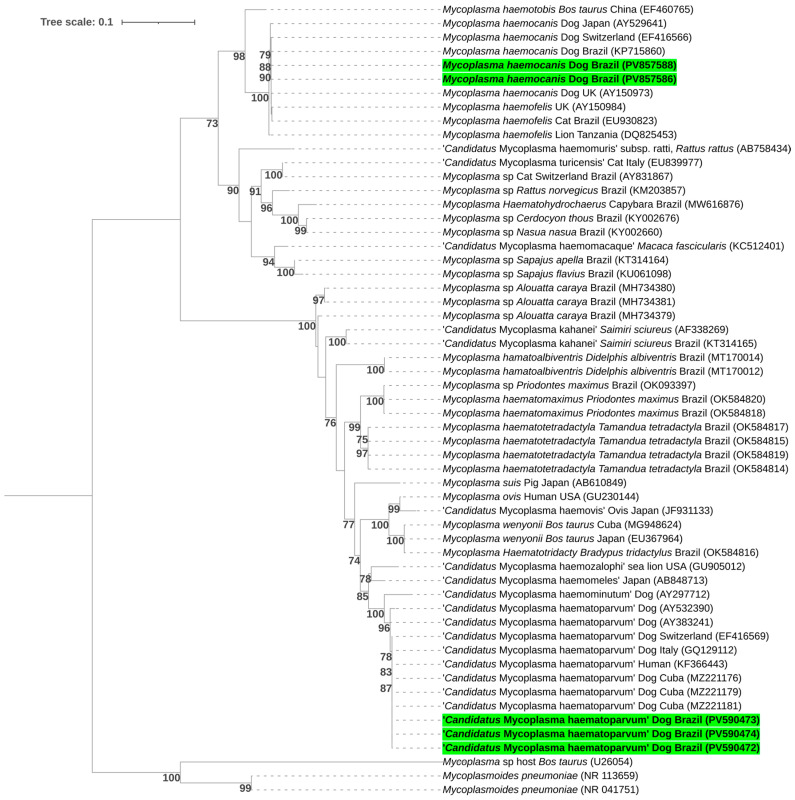
Phylogenetic tree inferred by the Maximum Likelihood method and GTR + I + G evolutionary model based on a 727 bp alignment of the 16S rRNA gene. Bootstrap values below 70% were not shown. Sequences obtained in this study are highlighted in green. Sequences shorter than 700 bp were excluded from the phylogenetic analysis. *Mycoplasmoides pneumoniae* and *Mycoplasma* sp. sequences were used as outgroups.

The phylogenetic analysis, inferred using the Maximum Likelihood method and the GTR + I + G evolutionary model, was based on the alignment of 727 bp of the 16S rRNA gene. It placed the obtained sequences in subclades of sequences of ‘*Candidatus* Mycoplasma haematoparvum’ previously detected in Cuba (MZ221181, MZ221179, and MZ221176), the USA (KF366443 and AY383241), Italy (GQ129112), Switzerland (EF416569), and France (AY532390), and of *Mycoplasma haemocanis* sequences previously obtained in Japan (AY529641), Switzerland (EF416566), Brazil (KP715860), and the UK (AY150973) (Figure 2).

**Figure 3:**
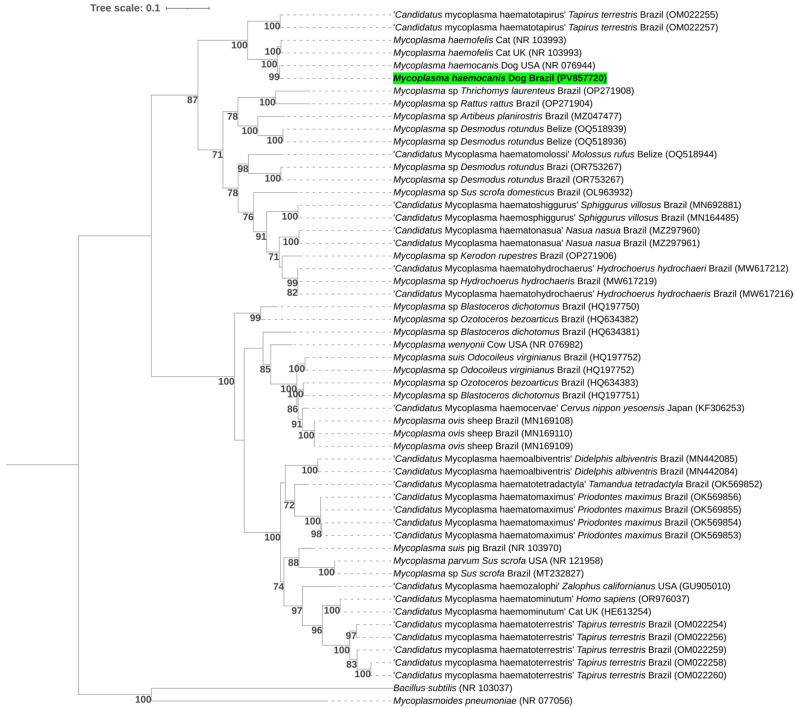
Phylogenetic tree inferred by the Maximum Likelihood method and GTR + I + G evolutionary model based on a 388 bp alignment of the 23S rRNA gene. Bootstrap values below 70% were not shown. Sequences obtained in this study are highlighted in green. Sequences shorter than 388 bp were excluded from the phylogenetic analysis. *Bacillus subtilis* and *Mycoplasmoides pneumoniae* sequences were used as outgroups.

The phylogenetic tree, inferred using the Maximum Likelihood method under the GTR + I + G substitution model and constructed from a 388 bp alignment of the 23S rRNA gene, grouped the sequences obtained into a subclade with a *Mycoplasma haemocanis* reference sequence previously described in the United States (NR_076944) (Figure 3).

### 3.5. Statistical Analysis

A total of 200 blood samples (100 from dogs undergoing routine clinical examinations and 100 from dog blood donors) were tested for the presence of hemopathogens. The prevalences ranged from 0% to 15% based on their groups (CL = dogs undergoing routine clinical examinations and CB = dog blood donors) and from 0% to 8.1% based on their sex (M = male and F = female). Table 2 and Table 3 represent the total number of analyzed animals, absolute and relative positivity for each analyzed agent and 95% confidence intervals based on the dog groups and sex, respectively.

Regarding the statistical analyses, when comparing the dogs’ groups (CL = dogs undergoing routine clinical examinations and CB = dog blood donors), significant differences were observed for *Anaplasma* spp. (*p* = 0.0289) and *Ehrlichia* spp. (*p* < 0.001), with all positive animals belonging to the group of dogs undergoing routine clinical examinations (CL). No significant differences were observed for *Bartonella* spp., *Babesia* spp., or *Mycoplasma* spp. (*p* > 0.05) (Table 4).

When comparing sex (M × F), none of the hemopathogens showed statistical significance (*p* > 0.05). Although only female dogs were positive for *Babesia* spp., the difference was not statistically significant (*p* = 0.078) (Table 5).

## 4. Discussion

In this study, while *E. canis*, *A. platys*, *B. vogeli*, and hemoplasmas were molecularly detected in blood samples from dogs submitted for routine veterinary clinical laboratory testing, only hemoplasma DNA was detected in blood samples from blood donor dogs. Conducting studies aimed at ensuring the safe use of blood components in Veterinary Medicine is essential.

In Brazil, few studies have been conducted aimed at detecting *Bartonella* spp. in blood donors. Pitassi et al. (2015) [55] and Drummond et al. (2023) [56] reported the detection of *B. henselae* and *Bartonella clarridgeiae* in human blood donors in Southeast Brazil.

In canine blood donors, reports are scarce worldwide and nonexistent in Brazil. *Bartonella* spp. DNA was not detected in a population of 262 canine blood donors in the United Kingdom using a commercial qPCR panel [57]. In Canada, *Bartonella* spp. was molecularly detected using a commercial qPCR panel in 0.081% of 6140 canine blood units [13].

The results found in our study, showing absence of *Bartonella* spp. detection in the tested samples, are consistent with the existing literature, which shows absence or low prevalence of *Bartonella* spp. in canine blood donors. Even with the combination of qPCR from blood samples, liquid culture (BAPGM enrichment medium) followed by qPCR, and plating on chocolate agar—which significantly increases the chance of detecting *Bartonella* spp. [58,59,60]—this agent was not detected in either group of dogs. Indeed, there are only two studies reporting the molecular evidence of *Bartonella* spp. in dogs in Brazil. Diniz et al. (2007) [61] found a molecular prevalence of 1% (2/198) and 0.5% (1/198) for *B. henselae* and *Bartonella vinsonii berkhoffii*, respectively, in a population of sick dogs from Brazil.

Although screening for *Bartonella* spp. is not routinely performed in Brazil for dog blood donors and no samples in our study tested positive, it is important to investigate the presence of this agent. A recent study conducted by our research group [62] detected *Bartonella henselae* in cat blood donors as well as in cats undergoing elective veterinary procedures. Since the true prevalence of *Bartonella* spp. in dogs in Brazil is still unknown and contact between dogs and cats is often frequent, the risks of transmission of this agent among donor dogs cannot be overlooked. A case of aortic valve endocarditis associated with *Bartonella clarridgeiae* was reported in a dog in Southeast Brazil [63].

Anemia and thrombocytopenia are amongst the most frequent hematological findings in dogs infected by *E. canis*, *A. platys*, and *B. vogeli* [64,65,66]. In this study, although DNA from these three tick-borne agents was detected in blood samples from the group of dogs undergoing clinical pathology tests regardless of clinical suspicion, such hemopathogens were not molecularly detected in blood samples from dogs registered as blood donors. Screening based on clinical evaluation, complete blood count, serum biochemistry (urea, creatinine, and total protein), and serological testing may justify the absence of detection of these agents in the blood donor group, since this screening would have revealed the main hematological alterations caused by these three tick-borne agents as well as the presence of anti-*Ehrlichia* spp. and anti-*Anaplasma* spp. antibodies, consequently leading to the exclusion of these dogs from the donor registry. Therefore, it is recommended that screening protocols be performed before each blood donation [67]. Thus, the adopted screening protocols used herein appeared to be effective in substantially reducing the risk of pathogen transmission. It should be noted, however, that the serological test used for screening in this study can detect exposure to only two of the agents investigated (*Ehrlichia* spp. and *Anaplasma* spp.).

However, it is important to highlight that chronically infected dogs with *Babesia* spp. may be asymptomatic and without significant hematological alterations, with parasitemia below the PCR detection threshold, resulting in false-negative results [68,69]. Likewise, infections by *Ehrlichia* spp. and *Anaplasma* spp. may present nonspecific or even absent hematological changes [70,71,72]. Additionally, the absence of detection of anti-*Ehrlichia* spp. and anti-*Anaplasma* spp. antibodies in serological screening may indicate acute phase infection in which antibodies are not yet detectable in blood samples. Therefore, although screening based on clinical and serological evaluations appeared to be effective for most dogs in the blood donor group in our study, PCR testing protocols should also be implemented.

Previously, Cruz et al. (2017) [9] found by PCR positivity of 25.7% (17/66) for *E. canis*, 9.1% (6/66) for *A. platys*, and 1.52% (1/66) for *B. vogeli* in dogs referred as candidate blood donors in the state of Mato Grosso, central-western Brazil. Nury et al. (2021) [13] detected antibodies against *Anaplasma phagocytophilum*/*A. platys* in 10 (0.56%) of 1779 blood units in Canada. In the same study, using a commercial qPCR panel, positivity for *A. phagocytophilum* was found in 4 (0.065%) of 6140 blood units.

Since *E. canis*, *A. platys*, and *B. vogeli* can cause similar clinical presentations, molecular diagnosis is an important tool to guide the veterinarian in determining the correct diagnosis and rapid treatment. On the other hand, hemotropic mycoplasmas have greater clinical relevance when associated with coinfections and immunocompromised dogs. The few reports of detection of these agents associated with clinical alterations occurred in splenectomized dogs, causing anemic conditions [73,74]. *Mycoplasma haemocanis* is the most common species of hemotropic mycoplasmas in dogs, followed by ‘*Candidatus* Mycoplasma haematoparvum’, the former being potentially more pathogenic [75].

In this context, it is important to highlight that ‘*Candidatus* Mycoplasma haematoparvum’ and *Mycoplasma haemocanis* were detected in blood samples from both group of dogs studied herein. According to the ABVHMT Technical Note No. 3 [6], the criteria to ensure the safety of donors and recipients include assessment of age, weight, temperament, clinical assessment, medication and procedure use, hematological and biochemical analyses, as well as screening for selected pathogens. The clinical and laboratory screening performed on blood donor dogs would initially consider them eligible for donation, since no agents from the genera *Ehrlichia*, *Anaplasma*, *Babesia*, or *Bartonella* were detected. However, this study shows that these hemoplasma species circulate “silently” in apparently healthy dogs. Previously, molecular occurrence rates of 0.6% (3/509) for *M. haemocanis* and 0.8% (4/509) for ‘*Candidatus* Mycoplasma haematoparvum’ have been reported in dogs sampled in the USA, with one *M. haemocanis* positive dog belonging to the apparently healthy group, and the remainder belonging to a diseased group [76].

The detection of ‘*Candidatus* Mycoplasma haematoparvum’ and *M. haemocanis* in blood samples from canine blood donors in this study, together with molecular evidence of the persistence of *M. haemocanis* in erythrocytes of dogs in stored blood bags [14], reinforces the importance of screening in blood donor dogs and canine blood bags. It is also important to emphasize that the careful selection of suitable donor candidates provides greater safety, as our results showed that the diversity of identified pathogens was considerably lower in pre-selected blood dogs, compared to dogs undergoing routine clinical examinations. In addition to the potential anemic condition caused or aggravated by these agents, hemoplasmas may also act as cofactors in the progression of retroviral, neoplastic, and immune-mediated diseases [77].

Studies have reported that the development of anemia in dogs infected with *Mycoplasma* spp. may take several weeks [11,78,79]; however, in acute cases, especially in immunocompromised and splenectomized dogs, rapid development of anemia may occur [80]. Thus, transfusing a potentially anemia-inducing agent—such as ‘*Candidatus* Mycoplasma haematoparvum’ and *M. haemocanis*—into dogs that are already anemic may exacerbate the existing anemia. Transfused erythrocytes could be parasitized and destroyed, impairing the beneficial effect of the transfusion for the patient. Furthermore, because anemia often develops only weeks after infection, the agent could cause new anemia episodes after patient stabilization, making them carriers and new sources of infection.

Although the occurrence of *E. canis*, *A. platys*, and *B. vogeli* has been extensively studied in Brazil [39,81,82,83,84,85,86], the same is not observed for hemoplasmas. Recently, Kmetiuk et al. (2025) [87] detected hemoplasma DNA in 23/644 (3.6%) humans from ten indigenous communities in southern and southeastern Brazil, with 3/644 (0.5%) of these individuals positive for *M. haemocanis*. For dogs, positivity was 21.9% (91/416), with 59.3% (54/91) for *M. haemocanis*, 29.7% (27/91) for ‘*Candidatus* M. haematoparvum’, and 11.0% (10/91) for both. Positivity of 94/159 (59.1%) for hemoplasmas was reported in hunting dogs from southern and midwestern Brazil [88]. In southern Brazil, Valle et al. (2014) [89] found a positivity of 5.1% (17/331) for *M. haemocanis* and 1.8% (6/331) for ‘*Candidatus* Mycoplasma haemominutum-like’ in blood samples from dogs attending a veterinary hospital. When evaluating hemoplasma presence by qPCR in humans, dogs, and horses in a rural settlement highly exposed to tick bites in southern Brazil, Vieira et al. (2015) [90] found positivity in 59/132 (44.7%) dogs, of which 20.3% were positive for ‘*C.* M. haematoparvum’, and in 1% (1/100) of humans. Kmetiuk et al. (2025) [91] detected 12/208 (5.8%) individuals from a quilombola community in Paraná State, southern Brazil, positive for hemoplasmas; *M. haemocanis* was confirmed in two sequenced samples. Among the dogs belonging to these individuals, 19/100 (19.0%) tested positive; 16/19 (84.2%) were positive for ‘*Candidatus* M. haematoparvum’, 7/19 (36.8%) for *M. haemocanis*, and 4/19 (21.0%) for both agents. Anemia was found among one human and three dogs positive for hemoplasmas.

It is worth pointing out that the *Tm* (“melting temperature”) values obtained in this study in positive samples for ‘*Candidatus* M. haematoparvum’ (77.0–77.5 °C) and *M. haemocanis* (78.0–78.5 °C) differed from the *Tm* values reported by Willi et al. (2009) [40] (73.0–74.0 °C and 75.0 °C, respectively), which may reflect variation in the 16S rRNA hemoplasma genotypes circulating in Brazil compared to those detected in the study by Willi et al. (2009) [40].

Guidelines on the proper execution of blood transfusions in dogs have been developed in different countries. In some cases, these guidelines are established by governmental bodies, such as those set by the Department of Animal Husbandry & Dairying (DAHD) in India. However, in many countries, there are no official or mandatory regulations for canine blood transfusion practices. In such cases, recommendations may be provided by specialized organizations, such as the American College of Veterinary Internal Medicine (ACVIM), the Canadian Veterinary Medical Association (CVMA), and the British Small Animal Veterinary Association (BSAVA).

In Brazil, there is no specific or mandatory regulation for canine blood donation. However, the guidelines of the ABVHMT (Brazilian Association of Veterinary Hematology and Transfusion Medicine), through Technical Note No. 3 [6], recommend screening for: (i) *Babesia vogeli*, *Ehrlichia* spp., *Anaplasma* spp., and *Leishmania infantum* by qPCR; (ii) *Brucella* spp. by qPCR in breeding animals; and (iii) other agents according to the epidemiological situation of each region. These guidelines are recognized and widely applied by institutions and professionals directly involved in canine transfusion practice.

Studies investigating a wide range of agents in canine blood donors or candidate dogs to blood donation are scarce in Brazil [9]. In the present study, we screened canine blood donors for several vector-borne pathogens. To the best of authors’ knowledge, this is the first report of hemoplasma detection in dog blood donors from Brazil.

Future studies should be conducted to identify the real prevalence of these agents in dog blood donors in Brazil. According to Wardrop et al. (2005) [92], screening of individual blood units for infectious disease agents should be considered the gold standard and, despite difficulties, such as higher costs and response time, it is a model that should be encouraged. Based on this, despite existing limitations, screening for infectious disease agents performed on blood samples collected at different times from the same animal may be a good strategy to ensure greater safety and reduce the risk of false negatives due to parasitemia fluctuations for the agents under study.

From a One Health perspective, the zoonotic potential of *E. canis*, *A. platys*, ‘*Candidatus* M. haematoparvum’, and *M. haemocanis* cannot be neglected. A new genotype of *E. canis* was molecularly detected in human blood donors in Costa Rica [93]. Coinfection with *B. henselae*, *A. platys*, and ‘*Candidatus* M. haematoparvum’ was detected in a veterinarian with a history of exposure to hematophagous arthropods and bites and scratches from various animals including dogs, cats, horses, birds, reptiles, rabbits, and rodents, presenting a complex and debilitating chronic clinical condition with neurological, musculoskeletal, cardiovascular, and autonomic manifestations [94]. More recently, *M. haemocanis* was detected in both indigenous and quilombola communities from Brazil [87,91]. These findings reinforce the zoonotic potential of hemopathogens circulating in dogs, highlighting the close relationship between human and animal health, both interconnected through the shared environment in which they live. In practice, this emphasizes the importance of continuous surveillance of the occurrence of these agents in dogs, allowing the extrapolation of risks to human and environmental health as a whole, and reinforcing key concepts within the One Health framework.

The findings of this study have direct implications for canine blood transfusion practices. Hemoplasma species were detected in both groups of dogs, with no statistically significant differences in prevalence, suggesting that these agents may occur subclinically even in carefully screened blood donors. This underscores the importance of incorporating molecular techniques into screening protocols to improve the detection of silent infections and ensure pathogen-free blood. On the other hand, the absence of *Ehrlichia* spp., *Anaplasma* spp, *Babesia* spp., and *Bartonella* spp. in dog blood donors, and the detection of *Anaplasma* spp. and *Ehrlichia* spp. in the group of dogs undergoing routine clinical examinations, showing a statistically significant difference compared to the group of blood donor dogs, demonstrate that the careful selection of donors, combined with serological and molecular testing, can substantially reduce the risk of hemopathogens transmission. Therefore, the adoption of an integrated screening approach should be considered the standard to ensure greater safety in veterinary blood transfusions.

Some limitations of this study include the small sample size for each group (100), the short sampling period (January to March 2023), the restricted study area (Federal District, Brazil), the absence of tick identification, and the lack of clinical correlation. Although it is desirable that the seasonal dynamics of vectors should be considered when evaluating the occurrence of vector-borne agents in dogs, the tick *Rhipicephalus sanguineus* sensu lato, one of the most important tick species infesting dogs in Brazil, does not appear to have its occurrence influenced by seasonality. According to Louly et al. (2007) [95], *R. sanguineus* sensu lato can complete up to four generations per year, showing population peaks throughout all seasons. Thus, although the sampling period was limited, it is unlikely that seasonality significantly influenced pathogen detection. Although tick collection was not performed in this study, the year-round presence of *R. sanguineus* in the Central-West region of Brazil, as suggested by Louly et al. (2007) [95], may contribute to the silent circulation of vector-borne pathogens in dogs, favoring the maintenance of asymptomatic animals as possible new sources of infection. In future studies, strategies to overcome these limitations should be considered in order to fill existing gaps in the knowledge regarding the presence of hemopathogens in canine blood donors.

## 5. Conclusions

This study molecularly detected the occurrence of *E. canis*, *A. platys*, ‘*Candidatus* M. haematoparvum’, and *M. haemocanis* in dogs undergoing diagnostic screening in a clinical pathology laboratory, evidencing the presence of these agents in Midwestern Brazil and demonstrating the importance of molecular assays for the detection of infectious disease agents in dogs, enabling rapid treatment initiation. This is the first study reporting the detection of hemotropic mycoplasmas in canine blood donors in Brazil. By including both dogs submitted for routine clinical examinations and registered blood donor dogs, this work provides relevant information on the silent circulation of these agents in dogs, indicating the need for continuous surveillance. Moreover, it shows that veterinarians should, in future procedures, adopt the combination of molecular and serological screening for hemopathogens for the careful selection of donors—measures that are essential to ensure transfusion safety, prevent the worsening of clinical conditions in recipient dogs, and avoid the emergence of new sources of infection.

## Figures and Tables

**Table 1:** Results of BLASTn analysis of *Babesia* spp., *Anaplasma* spp., *Ehrlichia* spp., and hemoplasma sequences obtained from blood samples of dogs submitted for routine analysis at a commercial clinical laboratory and from dogs registered as blood donors at a veterinary blood bank in midwestern Brazil.

GenBank Accession Number	Group	Target Gene	Sequence Length (bp)	Query Coverage	E-Value	Closest Match in GenBank	Host/Country
PV603924	Clinical routine	18S rRNA	776	100%	0.0	99.74%—*Babesia vogeli* (MK881089)	Dog/China
PV603925	Clinical routine	18S rRNA	658	100%	0.0	100%—*Babesia vogeli* (MN823199.1)	Dog/Brazil
PV608394	Clinical routine	*cox-1*	515	100%	0.0	100%—*Babesia vogeli* (KC207825)	Dog/USA
PV608395	Clinical routine	*cox-1*	523	100%	0.0	100%—*Babesia vogeli* (MZ577094)	Dog/Brazil
PV608396	Clinical routine	*cox-1*	584	100%	0.0	100%—*Babesia vogeli* (KC207825)	Dog/USA
PV608397	Clinical routine	*hsp70*	633	100%	0.0	100.00%—*Babesia vogeli* (AB248733)	Dog/Japan
PV608398	Clinical routine	*hsp70*	602	100%	0.0	98.69%—*Babesia vogeli* (AB248733)	Dog/Japan
PV608399	Clinical routine	*dsb*	308	100%	6 × 10^−158^	100% *Ehrlichia canis* (MT005829)	*Rhipicephalus linnaei*/Australia
PV575161	Clinical routine	16S rRNA	482	100%	0.0	100% *Anaplasma platys* (MK814414)	Dog/South Africa
PV575162	Clinical routine	16S rRNA	513	100%	0.0	100% *Anaplasma platys* (MK814414)	Dog/South Africa
PV590472	Clinical routine	16S rRNA	1027	100%	0.0	99.52%—‘*Candidatus* Mycoplasma haematoparvum’ (AY383241)	Dog/USA
PV590473	Blood donor	16S rRNA	978	100%	0.0	100%—‘*Candidatus* Mycoplasma haematoparvum’ (MZ221179)	Dog/Cuba
PV590474	Blood donor	16S rRNA	897	100%	0.0	100%—‘*Candidatus* Mycoplasma haematoparvum’ (MZ221181)	Dog/Cuba
PV857586	Clinical routine	16S rRNA	739	100%	0.0	100%—*Mycoplasma haemocanis* (GQ129116)	Dog/Italy
PV857587	Blood donor	16S rRNA	423	100%	0.0	100%—*Mycoplasma haemocanis* (MT816510)	Dog/Portugal
PV857588	Blood donor	16S rRNA	701	100%	0.0	100%—*Mycoplasma haemocanis* (GQ129116)	Dog/Italy
PV857719	Clinical routine	23S rRNA	256	100%	4 × 10^−129^	100%—*Mycoplasma haemocanis* (NR_076944)	Dog/USA
PV857720	Blood donor	23S rRNA	435	100%	0.0	99.77%—*Mycoplasma haemocanis* (NR_076944)	Dog/USA

**Table 2:** Molecular occurrence of hemopathogens according to the analyzed groups of dogs. *N* = total number of animals.

Hemopathogen	Dog Group	*N*	Positive	Prevalence (%)	CI_Low	CI_High
*Anaplasma* spp.	CL	100	6	6	0.028	0.125
	CB	100	0	0	0	0.037
*Babesia* spp.	CL	100	3	3	0.010	0.085
	CB	100	0	0	0	0.037
*Bartonella* spp.	CL	100	0	0	0	0.037
	CB	100	0	0	0	0.037
*Ehrlichia* spp.	CL	100	15	15	0.093	0.233
	CB	100	0	0	0	0.037
*Mycoplasma* spp.	CL	100	2	2	0.006	0.070
	CB	100	5	5	0.022	0.112

CL = dogs undergoing routine clinical examinations; CB = dog blood donors; CI = confidence interval.

**Table 3:** Molecular occurrence of hemopathogens according to the analyzed dogs’ sex. *N* = total number of animals.

Hemopathogen	Dog Sex	*N*	Positive	Prevalence (%)	CI_Low	CI_High
*Anaplasma* spp.	M	114	4	3.51	0.014	0.087
	F	86	2	2.33	0.006	0.081
*Babesia* spp.	M	114	0	0	0	0.033
	F	86	3	3.49	0.012	0.098
*Bartonella* spp.	M	114	0	0	0	0.033
	F	86	0	0	0	0.043
*Ehrlichia* spp.	M	114	8	7.02	0.036	0.132
	F	86	7	8.14	0.040	0.159
*Mycoplasma* spp.	M	114	3	2.63	0.009	0.075
	F	86	4	4.65	0.018	0.114

CI = confidence interval.

**Table 4:** Comparison of positive results for hemopathogens according to the analyzed groups of dogs. OR = odds ratio; *p*-value was considered significant when *p* < 0.05.

Hemopathogen	*p*_Value	Odds_Ratio	CI_Low	CI_High
*Anaplasma* spp.	0.02893028	0	0	0.826
*Babesia* spp.	0.24623116	0	0	2.406
*Bartonella* spp.	1	0	0	Inf
*Ehrlichia* spp.	3.4634 × 10^−5^	0	0	0.248
*Mycoplasma* spp.	0.44475403	2.56747153	0.408	27.586

CI = confidence interval.

**Table 5:** Comparison of positive results for hemopathogens and sex of the analyzed dogs. OR = odds ratio; *p*-value was considered significant when *p* < 0.05.

Hemopathogen	*p*_Value	Odds_Ratio	CI_Low	CI_High
*Anaplasma* spp.	0.70137731	0.65609672	0.058	4.702
*Babesia* spp.	0.0779199	Inf	0.553	Inf
*Bartonella* spp.	1	0	0	Inf
*Ehrlichia* spp.	0.79169103	1.17309712	0.346	3.876
*Mycoplasma* spp.	0.46605801	1.7994358	0.296	12.621

CI = confidence interval.

## Data Availability

The sequences obtained in this study have been submitted to the NCBI GenBank (https://www.ncbi.nlm.nih.gov/genbank/, accessed on 4 May 2025), and can be retrieved using the following accession numbers: PV603924, PV603925, PV608394, PV608395, PV608396, PV608397, PV608398, PV608399, PV575161, PV575162, PV590472, PV590473, PV590474, PV857586, PV857587, PV857588, PV857719, PV857720.

## Supplementary Materials

The following supporting information can be downloaded at: https://www.mdpi.com/article/10.3390/pathogens14111180/s1, Supplementary Material S1: Primers and PCR conditions for *Bartonella* spp., piroplasmids, *Ehrlichia* spp., *Anaplasma* spp., and hemotropic *Mycoplasma* spp. detection.
