# Supplementary material for: Molecular Survey of Hemopathogens in Dogs, Including Blood Donors, from Central-Western Brazil
Source: Pathogens. 2025 Nov 18;14(11):1180. doi: 10.3390/pathogens14111180 (PMC12655668; doi:10.3390/pathogens14111180)
Supplement: Supplementary file 1 [file pathogens-14-01180-s001.zip › pathogens-3962023-supplementary.pdf]

Supplementary Material S1. Primers and PCR conditions for *Bartonella* spp., piroplasmids, *Ehrlichia* spp., *Anaplasma* spp., and hemotropic *Mycoplasma* spp. detection.

Agent	Gene/ name primer	Primers sequences	Size (bp)	Thermal sequences	Reference
**Endogenous gene**	***gapdh*** -GAPDHF -GAPDHR	5'TCCAACACCACCACTGAGATCGGAC -3' 5'- GTGAGAAGAAATCGGACTGGCC3'	400	95°C for 5 min; 35 cycles: 95°C for 15s, 50°Cfor 30s and 72°C for 30s; 72ºC for 5 min	Birkenheuer et al. (2003)
**Piroplasmids (screening -nPCR)**	18S rRNA 1st Round -BTF1 -BTR1 2nd Round -BTF2 -BTR2	1st Round 5'GGCTCATTACAACAGTTATAG-3' 5'CCCAAAGACTTTGATTTCTCTC-3' 2nd Round 5'CCGTGCTAATTGTAGGGCTAATAC-3' 5'GGACTACGACGGTATCTGATCG-3'	800	94°C for 3 min, 58ºC for 1 min, 72ºC for 2 min; 45 cycles: 94°C for 30s, 58°C for20s and 72°C for 30s; 72°C for 7 min Annealing temperature 2nd round =62ºC	Jefferies et al. (2007)
**Piroplasmids (characterization)**	18S rRNA -Nbab_1F -18SApiR	5'-AAGCCATGCATGTCTAAGTATAAGCTTTT-3' 5'-GGATCACTCGATCGGTAGGAG-3'	1500	95°C for 5 min; 50 cycles: 95°C for 30s, 60°C for 30s and 72°C for 2min; 72°C for 5 min	Greay et al. (2018)
**Piroplasmids (characterization - nPCR)**	18S rRNA 1st Round -Piro 0F -Piro 6R 2nd Round -Piro 1F -Piro 5.5R	1st Round 5'-GCCAGTAGTCATATGCTTGTGTTA-3' 5'-CTCCTTCCTYTAAGTGATAAGGTTCAC-3' 2nd Round 5'-CCATGCATGTCTWAGTAYAARCTTTTA-3' 5'-CCTYTAAGTGATAAGGTTCACAAAACTT-3'	1500	95°C for 3 min; 33 cycles: 95°C for 1min, 59°C for 1min and 72°C for 2min; 72°C for 10 min	Kawabuch et al.,2005
**Piroplasmids (characterization- nPCR)**	*cox-1* 1st Round -Bab_for1 -Bab_Rev1 2nd Round -Bab_for2 -Bab_rev2	1st Round 5'ATWGGATTYTATATGAGTAT3' 5'ATAATCWGGWATYCTCCTTGG3' 2nd Round 5'TCTCTWCATGGWTTAATTATGATAT3' 5'TAGCTCCAATTGAHARWACAAAGTG3'	924	95ºC for 1 min; 35 cycles; 95ºC for 15s, 45ºC for 30s and 72ºC for 1 min 72ºC for 10 min Annealing temperature 2nd round =49ºC	Corduneanu et al. (2017)
**Piroplasmids (characterization)**	*cox-3* -Cox3F -Cox3R	5'-ACTGTCAGCTAAAACGTATC-3' 5'-ACAGGATTAGATACCCTGG-3'	600	94°C for 5 min; 50 cycles: 94°C for 20s, 55°Cfor 30s and 68°C for 45s; 72ºC for 7 min	Schreeg et al. (2016); Barbosa et al.(2019)
**Piroplasmids (characterization)**	*hsp70* -Hsp70F1 -Hsp70R2	5'-CATGAAGCACTGGCCHTTCAA- 3' 5'-GBAGGTTGTTGTCCTTVGTCAT-3'	1056	95ºc for 5 min; 35 cycles: 95ºC for 15s, 60ºC for 30s and 72ºC for 30s; 72ºC for 5 min	Soares et al. (2011)

**Bartonella spp. (screening - qPCR)**	16S-23S ITS<br>BsppITS325S Forward<br>543as Reverse<br>BsppITS500	5’ – CTTCAGATGATGATCCCAAGCCTTCTGGCG – 3’<br>5’ – AATTGGTGGGCCTGGGAGGACTTG – 3’<br>5’ – [6FAM]GTTAGAGCGCGCGCTTGATAAG[BHQ1] – 3’	243	95ºC for 3min; 45 cycles of 94ºC for 10s, 66ºC for 10s, 72ºC for 10s and plate read	(Breitschwerdt & Maggi, 2019; Oteo et al., 2017)
***Ehrlichia* spp. *Anaplasma* spp. (screening) mulitplex qPCR**	(*groEL*)<br>F-Ehr<br>R-Ehr<br>TET-<br>F-Anap<br>R-Anap<br>Cy5-	5′- TTATCGTTACATTGAGAAGC - 3′),<br>5′- GATATAAAGTTATTAAAAGTATAAAGC -3′)<br>5′-CATTGGCTCTTGCTATTGCTAAT -3′[BHQ2a-Q]<br>5′- GCGAGCATAATTACTCAGAG-3′),<br>5′- CAGTATGGAGCATGTAGTAG -3′) e<br>5′- CCACCTTATCATTACACTGAGACG -3′[BHQ2a-Q]	83	95 °C for 3 min,<br>40 cycles: 95 °C for 10 s and 52.7 °C for 30 s.	Benevenute et al., 2017
***Ehrlichia* spp. (screening) qPCR**	(*dsb* gene)	5′-TTG CAA AAT GAT GTC TGA AGA TAT GAA ACA -3′<br>5′-GCT GCT CCA CCA ATA AAT GTA TCY CCTA-3′<br>[5′ FAM AGC TAG TGC TGC TTG GGC AAC TTT GAG TGA A-[BHQ-1–3′]	378	95 °C for 5 min,<br>40 cycles: 95 °C for 15 s and 60 °C for 1 min	Doyle et al., 2005
***Anaplasma* spp. (characterization)**	16S rRNA<br>-gE3a<br>-gE10R<br>-gE2<br>-gE9f	5′- CACATGCAAGTCGAACGGATTATTC-3′<br>5′- TTCCGTTAAGAAGGATCTAATCTCC′-3′<br>5′- GGCAGTATTAAAAGCAGCTCCAGG-3′<br>5′-AACGGATTATTCTTTATAGCTTGCT-3′	548	94°C for 5 min;<br>40 cycles: 94°C for 30s, 55°C for 30s and 72°C for 1 min;<br>72°C for 5 min	Massung et al., 1998
***Ehrlichia* spp. (characterization)**	*dsb*<br>-dsb-330<br>-dsb-728	5’-GATGATGTCTGAAGATATGAAACAAAT-3’<br>5’- CTGCTCGTCTATTTTACTTCTTAA,AGT 3’	409	95°C for 2 min;<br>50 cycles: 95°C for 30s, 55°C for 30s and 72°C for 1min;<br>72°C for 5 min	Doyle et al., 2005
***Hemoplasmas (screening) qPCR**	16SrRNA<br>SYBR_For<br>SYBR_Rev1<br>SYBR_Rev2	5′-AGCAATRCCATGTGAACGATGAA-3′<br>5′-TGCACATAGTTTTGCTGTCACTT-3′<br>5′-GCTGGCACATAGTTAGCTGTCACT-3′	-	50°C for 2 min and 95°C for 10 min<br>40 cycles: 95 °C for 15 s, 60 °C for 1 min	Willi et al., 2009
**Hemoplasmas (characterization)**	23S rRNA<br>-23S HAEMO F<br>-23S HAEMO R	5′- TGAGGGAAAGAGCCCAGAC - 3′<br>5′- GGACAGAATTTACCTGACAAGG - 3′	800	94°C for 3 min;<br>35 cycles: 94°C for 30s, 54°C for 30s and 72°C for 1 min;<br>72°C for 10 min	Mongruel et al., 2020
**Hemoplasmas (characterization)**	16S rRNA<br>-HEMOF1<br>-HEMOR2<br>-41SF<br>-938aSR	5′ AGAGTTTGATCCTGGCTCAG – 3’<br>5′ - TACCTTGTTACGACTTAACT - 3′<br>5′- GYATGCMTAAYACATGCAAGTCGARCG -3′<br>5′ - CTCCACCACTTGTTCAGGTCCCCGTC - 3′	1200	95°C for 5 min;<br>35 cycles: 95°C for 30s, 57°C for 30s and 72°C for 1min;<br>72°C for 10 min	Di Cataldo et al., 2020
**Hemoplasmas (characterization)**	16S rRNA<br>Myco16S-322s<br>Myco16S-938as	5′-GCC CAT ATT CCT ACG GGA AGC AGC AGT-3′<br>5′-CTC CAC CAC TTG TTC AGG TCC CCG TC-3′	620	95 °C for 2 min;<br>55 cycles: 94 °C for 15 s, 68 °C for 10 s and 72 °C for 15 s;<br>72 °C por 30 s	Maggi et al., 2013
	Myco16S-938as and HemMycop16S-41s<br>Myco16S-938as and HemMycop16S-1420as	5′-GYA TGC MTA AYA CAT GCA AGT CGA RCG-3′<br>5′-GTT TGA CGG GCG GTG TGT ACA AGA CC-3′	1380		

*The two reverse primers (SYBR_Rev1 and SYBR_Rev2) were used as a 1:1 mixture in the SYBR green PCR.
